# Integration of Multivariate Statistical Control Chart and Machine Learning to Identify the Abnormal Process Parameters for Polylactide with Glass Fiber Composites in Injection Molding; Part I: The Processing Parameter Optimization for Multiple Qualities of Polylactide/Glass Fiber Composites in Injection Molding

**DOI:** 10.3390/polym15143018

**Published:** 2023-07-12

**Authors:** Chi-Hao Hsiao, Chang-Chiun Huang, Chung-Feng Jeffrey Kuo, Naveed Ahmad

**Affiliations:** Department of Materials Science and Engineering, National Taiwan University of Science and Technology, Taipei 10607, Taiwan; m10104206@mail.ntust.edu.tw (C.-H.H.); huangcc@mail.ntust.edu.tw (C.-C.H.); d10804833@mail.ntust.edu.tw (N.A.)

**Keywords:** polylactide, glass fiber, injection molding, Taguchi method, principal component analysis, data envelopment analysis

## Abstract

This paper discusses the mixing of polylactide (PLA) and glass fiber which use injection molding to produce a functional composite material with glass fiber properties. The injection molding process explores the influence of glass fiber ratio, melt temperature, injection speed, packing pressure, packing time and cooling time on the mechanical properties of composite. Using the orthogonal table planning experiment of the Taguchi method, the optimal parameter level combination of a single quality process is obtained through main effect analysis (MEA) and Analysis of variance (ANOVA). Then, the optimal parameter level combination of multiple qualities is obtained through principal component analysis (PCA) and data envelopment analysis (DEA), respectively. It is observed that if all the quality characteristics of tensile strength, hardness, impact strength and bending strength are considered at the same time, the optimal process conditions are glass fiber addition 20 wt %, melt temperature 185 °C, injection speed 80 mm/s, holding pressure 60 MPa, holding time 1 s and cooling time 15 s, and the corresponding mechanical properties are tensile strength 95.04 MPa, hardness 86.52 Shore D, impact strength 4.4408 J/cm^2^, bending strength 119.89 MPa. This study effectively enhances multiple qualities of PLA/GF composite.

## 1. Introduction

Nowadays, the importance of improving component mechanical and thermal qualities, incorporating them into production systems, and employing eco-friendly materials is rising [1]. On the contrary, every industry must manage its environmental impact from conception to final disposal. A material that is thermo-mechanically durable, lightweight, and biodegradable is required. The demand for poly(lactic acid) (PLA) composites reinforced with natural and synthetic fibers has surged as a result of this sectoral development [2].

PLA can be biodegradable, low-pollution and non-toxic, high mechanical strength, biocompatibility, application in the fields of medicine, packaging, and daily necessities, which can greatly reduce the impact of resources on environmental damage, and become a new type of polymer material with great development potential [3]. PLA polymers can be produced through direct lactic acid poly-condensation and also via ring-opening polymerization of lactide, a cyclic dimer of lactic acid [4]. Because the PLA products produced by traditional processing technology have a slow crystallization rate and low crystallinity in the molding process. PLA also has poor heat resistance, with a heat deflection temperature (HDT) of 55–65 °C, which seriously limits the application range of PLA at higher temperatures [5], such as disposable heat-resistant products, utensils and other food packaging containers [6]. PLA stands out with its eco-friendly properties so it has been recently more preferable to synthetic polymers in some sectors. There are some issues such as poor heat resistance of PLA, poor crystallization rate, deformation and material brittleness of disposable tableware during transportation, so, there is an urgent need to modify PLA to improve its heat resistance and all other properties as well. The use of biodegradable polymer PLA is limited, so it is necessary to enhance its properties through composite reinforcements.

Glass fiber (GF) is effectively to modify the substrate, and enhance the mechanical properties as well. After recycling, it can be reused and meets environmental protection requirements. It is a well-known reinforcement material in the composites sector as well [7]. Since it comprises inorganic components, it has excellent dimensional stability, transparency, and mechanical appropriateness [8]. SiO_2_, Al_2_O_3_, Fe_2_O_3_, CaO, MgO, Na_2_O, K_2_O, and BaO could be identified as the primary chemical constituents of glass fiber [9]. Because GF has a low thermal conductivity, it can dissipate heat similarly to asbestos and other organic fibers [10]. This makes it a popular insulation material in many industries. GF reinforced plastics continue to be the most prominent and dominant material in the industry, despite advancements and innovations in the plastic-composite field.

Wang et al. [11] showed not only GF but also enhanced crystallization led to the outstanding mechanical performance of PLA/GF composites. While GF shows heat treatment can remarkably improve thermal stability, in particular for PLA/GF composites. Sun et al. [12] presented that the defect in fiber GF reinforced PLA is significantly improved by modification and the mechanical properties are improved by about 40%. The main reason is the unification of the surface polarity of fibers and PLA, as well as the connection established by the functional groups. Meanwhile, the surface modification of GF/PLA composites can also improve their thermal and degradation properties. Wang et al. [13] demonstrated that the outstanding mechanical properties arises from the strengthening effect of the GF network skeleton that shows good bonding with PLA matrix. GF led to simultaneously enhanced strength, rigidness and toughness of PLA. Thermal analysis showed that GF led to increased heat deflection temperature of PLA. Leu et al. [14] used injection molding for PLA/maleated styrene-ethylene/butylene-styrene/organo-montmorillonite to improve the mechanical properties of composite materials. Ma et al. [15] discussed preparation and foaming extrusion behavior of PLA/polybutylene succinate (PBS)/montmorillonoid nanocomposite. The compatibility between PLA and PBS, and the elongation at break and impact strength of composite materials can be improved. Shen et al. [16] prepared biocomposite of PLA/reinforced hydroxyapatite (HA)/carbon fiber from hot pressing a prepreg which was manufactured by solvent impregnation process in order to improve composite mechanical properties. Bledzki et al. [17] used injection molding to make tensile and impact test pieces to verify the improved mechanical properties of composites of PLA man-made cellulose and abaca fibers. An et al. [18] investigated the cutting characteristics of GF reinforced plastics with respect to tool materials and geometries to improve tensile strength, impact and flexural strength, corrosion resistance and non-conductive properties. Bigg et al. [19] presented that GF can be effective increase the mechanical properties of materials such as strength and stiffness in thermoplastic composite materials. Jaszkiewicz et al. [20] applied GF/abaca fiber/man-made cellulose to prepare composite materials with PLA and polypropylene (PP) respectively, showing that GF can enhance the impact strength of PLA and PP.

PLA has excellent processing performance and can be processed by extrusion, film blowing and injection molding [21,22,23]. The extrusion process has some disadvantages because of nozzle radius limits, reduces the final quality, limits the accuracy and speed when compared to other processes, consistent pressure of material is required in order to increase quality of finish. In film blowing method, it is difficult to control accurately the thickness of a blown film and method is quite complicated as well, and there are a number of factors that can go wrong, the manufacturing cost for blown films is high and not environment-friendly.

Oppositely, the injection molding is advantageous as compared to other techniques because its low-waste process, it minimizes molding costs, highly repeatable way of production with high precision. Injection molding can produce a huge amount of parts per hour from a wide range of other materials, injection molding technology can limit the waste by recycling wherever possible, planning production runs to maximize efficiency, and conserving energy. It has become the most important production technology in polymer plastics and composite plastic materials [24,25]. The materials and process parameters are the important factors affecting product quality.

Therefore, this paper will discuss the mixing of PLA and glass fiber which use injection molding to produce a functional composite material with glass fiber properties. The influence of processing parameters will be discussed on various qualities through the injection molding process. The orthogonal table in Taguchi method will be used to plan the experiment. Through the MEA and ANOVA to obtain the process optimization parameters of a single quality. In response to the multiple quality characteristics of this study, PCA is applied to reduce the dimensionality of the relevant quality characteristics into independent linear combinations, and DEA method calculates the objective optimal weight of the original data to obtain relative efficiency. Afterwards, the optimal combination of processing parameter factor levels will be found and the confirmation experiments will be conducted to verify the optimized results. PLA based glass fibers that are added in higher content to produces a desirable characteristic so that the treated fibers produced desirable reinforcement effects.

## 2. Experimental Methodology

### 2.1. Injection Molding Process

Plastic injection molding is one of the most widely used plastic fabrication processes for plastic mass production with numerous shapes and complicated geometries. It has preliminarily been estimated that over 30% of the polymers that are processed as well as consumed are produced by the injection-molded process [26,27].

In the injection molding manufacturing, while there are a number of parameters that must be determined, some have been recognized as the important process parameters in relation to product quality. As the most popular plastic molding processing method at present, Bozzelli [28] proposed that melt temperature, injection pressure, injection speed and cooling time are important factors for plastic thin shell injection molding. Jansen et al. [29] pointed out that the impact on the shrinkage, the biggest ones are melt temperature, holding pressure and injection speed. Shokri et al. [30] showed that the properties of fiber-reinforced thermoplastic injection molding products depend on the influence of packing pressure on fiber orientation. Kamaruddin et al. [31] presented that melt temperature, high injection pressure, low packing pressure, long holding time and cooling time can effectively reduce the shrinkage behavior of injection molded products.

Kuo et al. [32] indicated that cooling time, mold temperature, melt temperature, injection speed, injection pressure, packing pressure, and packing time are the key factors for plastic LCD light-guide plates.

The related research concerning about the process factors include the mold temperature, the melt temperature, the packing pressure, the packing time and cooling time [26]. The current manufacturing application determining the injection molding process parameters involves a combination of the use of the machine operation handbook and accompany with the adaptations through trial and error from experienced plastic engineers [26]. In order to guarantee that the optimal process parameters have been selected, the demand to establish these optimal parameters has given rise to this research.

### 2.2. Process Optimization

In traditional experiments, when the process parameters increase, the number of experiments will increase. In order to solve this problem, Karna et al. [33] used the Taguchi method, the robust design of the orthogonal table, the S/N ratio and ANOVA to study the impact of process parameters on the product. Liu et al. [34] used Taguchi method to analyze the parameter optimization of thin shell parts in the injection molding process, showing that the melt temperature and injection pressure are the most important processing parameters. Ghani et al. [35] using the Taguchi method in the high-speed milling process, through the S/N ratio and ANOVA, the optimal process parameters are optimized. However, the Taguchi method is only suitable for the improvement of a single quality. In actual industry, it needs to be combined with other analysis methods to achieve the goal of multi-quality process optimization. For example, Su et al. [36] applied principal component analysis method to reduce the dimension and complexity and solved multi-quality problems. Antony [37] used the PCA method, combined with the quality loss function to effectively improve and take into account the effect of multi-quality. Shih et al. [38] presented the inert gas shielded welding process to weld the foamed aluminum plate. Taguchi method combined with the PCA method showing that the current, welding speed and the gap between the workpieces are important control factors in the process. The optimal parameters of the process could improve the multi-quality characteristics of the aluminum foam board. Jeyapaul et al. [39] aimed at the operation of the gear processing machine with six control factors. It showed that compared with Taguchi method, the genetic algorithm and DEA method are used for the optimal factor level combination S/N ratio of the qualities, and the total expected improvement is 4.1498 db and 11.2506 db, respectively. Al-Refaie et al. [40] studied the improvement of the quality of the hard disk drive with controllable factors. Compared with Taguchi method, when PCA method and DEA method used to optimize the quality process parameters, the total expected improvement of the optimal factor level combination S/N ratio are 4.1498 db and 11.2506 db, respectively

Therefore, this paper will use the Taguchi method, and combine with PCA and DEA to achieve the goal of the optimizing multiple qualities.

### 2.3. Materials

Manufacturer: Nytex Composites Co., Ltd. New Taipei City, Taiwan. Product number: GG-0010N (TY11512706, 10% Glass fiber), GG-0015N (TY11512707, 15% Glass fiber), GG-0020N (TY11512708, 20% Glass fiber).

The material properties are shown in Table 1.

### 2.4. Experimental Methodolofy

This section will introduce the injection molding, material analysis, possible reaction of the composite and experimental scheme.

#### 2.4.1. Injection Molding

Injection molding machines perform a wide range of mechanical movements with differing characteristics. Mold opening is a low-force high-speed movement, and mold closing a high-force low-speed movement. Plasticizing involves high torque and low rotational speed, while injection requires high force and medium speed. Injection molding machine consist of three major components i.e., (1) Screw motor drive (2) Reciprocating screw and barrel, (3) Heaters, thermocouple, and ring plunger.

**Figure 1:**
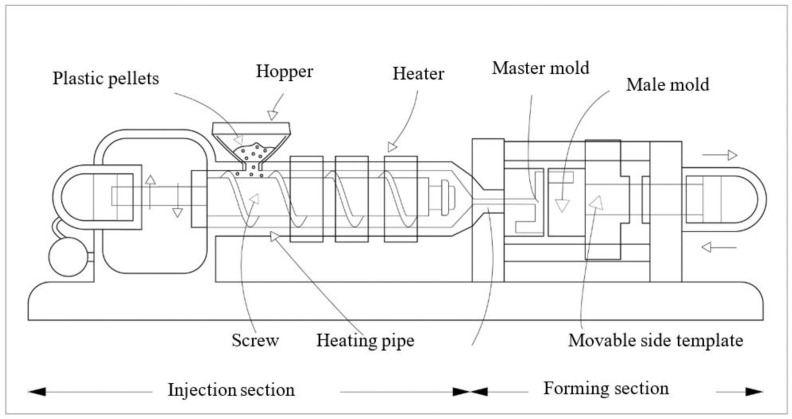
The operation principle of the injection molding machine.

**Figure 2:**
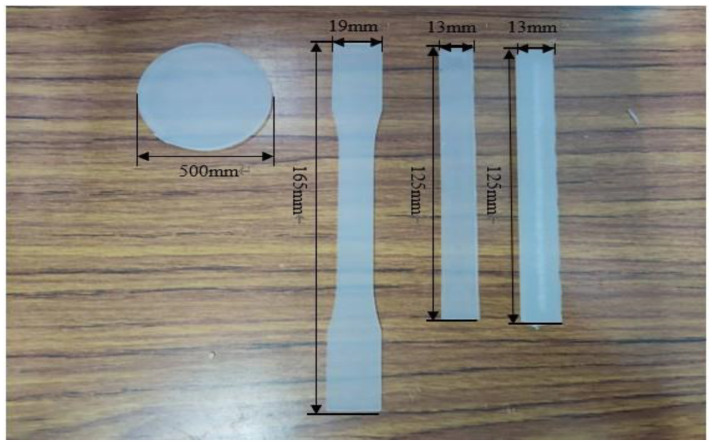
The injection samples.

The operation principle of the injection molding is very simple, where plastic material is heated above its melting point, resulting in the conversion of the solid polymer to a molten fluid with a reasonably low viscosity. It is then forced into a closed mold that defines the shape of the article to be produced. The operation elements are shown in Figure 1. The injection samples are shown in Figure 2.

The plastic material from the feeding hopper enters the barrel, mixed by the screw, sent to the front end of the heating tube along the spiral groove, and is heated by the peripheral heater. The screw rotates to fully mix the plastic material so that the plastic is in a molten state. When the screw rotates, the screw retreats due to the reaction force (back pressure) of the plastic material. At this time, use the limit switch to constrain the amount of retreat, stop the screw rotation at a certain position, then close the mold into the injection stage. Meanwhile, the hydraulic cylinder of the injection device exerts injection force on the screw, and the screw becomes an injection plunger. Under high pressure, the completely melted plastic material at the front end of the barrel is injected into the mold from the nozzle. After the material in the cavity cools down, the mold is opened and eject the finished product. The injection molding machine can form plastic products with complex shapes, precise dimensions or dense texture with metal inserts at one time.

#### 2.4.2. Materials Analysis

The instrument used is differential scanning calorimeter (DSC) for thermal properties. The instruments to measure mechanical properties such as tensile strength, Shore hardness, impact strength, and bending strength. The model used is MTS 810, the maximum displacement range: ±75 mm, the maximum test load: ±100 kN. Comply with ASTM D790 standard, observe the strength change of tension and bending. According to the ASTM D2204-00 standard, the composite material studied is more than 90 Shore A, using D type Shore hardness tester. The impact test is to determine the toughness of the material. The model of Izod impact testing machine used in this research is Yasuda Seiki N0158, which measures the impact energy of materials according to ASTM D256 standard.

**Figure 3:**
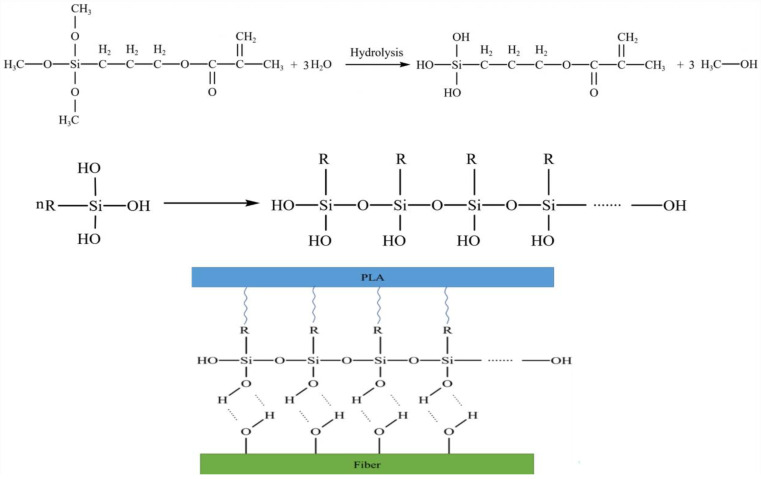
Possible reaction between polylactic acid (PLA) and glass fiber.

The possible reaction between PLA and GF to synthesize PLA/GF composite is given in Figure 3.

**Figure 4:**
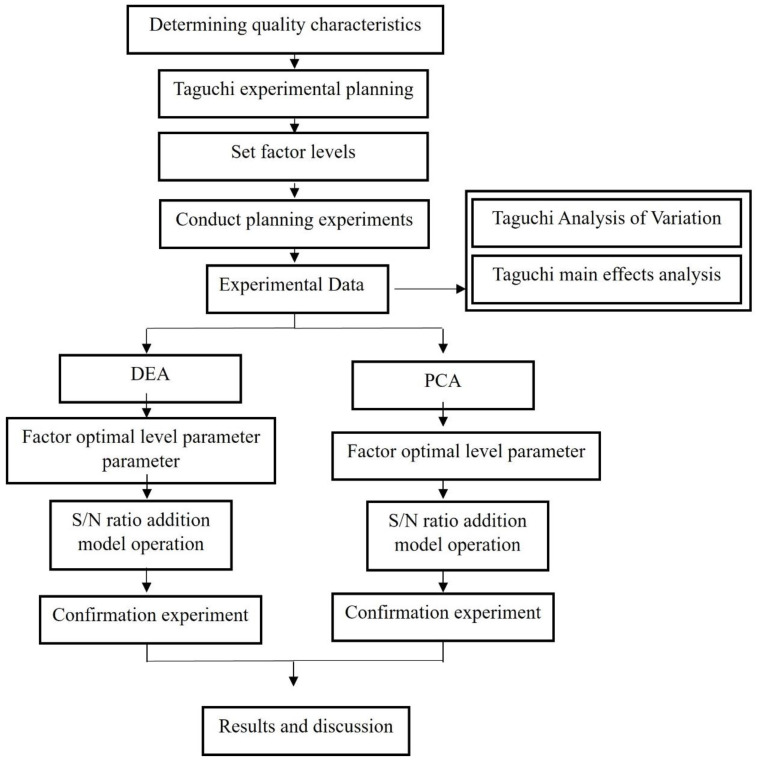
Scheme of experiment and processing.

A coupling agent versatile molecule, was employed to modify the fiber surface which generate a chemical bond between the siloxy group and the alkyl group. Silane coupling agents transformed fibers by a multi-step process that included bonding, condensation, and hydrolysis. The hydrolysis of siloxy groups resulted in the formation of silanol. The hydrophobicity of the molecule was increased by its ability to interact with the hydroxyl group of cellulose during the condensation process, and the opposite side of the molecule reacted with the PLA matrix to establish a bond (Figure 4). The enhancement of interfacial characteristics was credited for a boost in tensile strength and flexibility. Another purpose of silane is to serve as a surface protective layer by penetrating the pores of the fiber surface.

#### 2.4.3. Scheme of Experiment and Processing

In this section, the material properties of the composite material are analyzed to set up the range of processing parameters. The L_18_ orthogonal table is used to plan the experiment, combined with DEA and PCA respectively to achieve the optimization of multiple qualities. Then, the optimized parameter combination is implemented in the confirmation experiment to verify the feasibility and reproducibility of the optimized parameters. The planning process of this experiment is shown in Figure 4.

## 3. Taguchi and Other Statistical Techniques

This study uses PCA and DEA to optimize the process parameters of PLA and GF composites used in injection molding machines.

### 3.1. Taguchi Method [41]

#### 3.1.1. Orthogonal Table

The orthogonal table is expressed as L_a_ (b^c^) represents the orthogonal table, a is the column number (experiment times), b is the level number, and c is the row number.

#### 3.1.2. Signal-to-Noise (S/N) Ratio

The quality discussed in this study is the larger the mechanical properties of tensile strength, Shore hardness, impact strength, and bending strength, the larger the better (LTB). The S/N ratio of the maximum characteristic is defined as:

(1) $$S/N_{LTB}=-10{log}_{10}(\frac{1}{n}\sum_{i=1}^{n}\frac{1}{{y_{i}}^{2}})$$

where MSD is the mean square deviation from the target value, *n* is the total number of measurements, and $y_{i}$ is the quality measurement value.

#### 3.1.3. Main Effects Analysis (MEA)

Find the average response value of each factor level and the main effect value ΔFi from the experimental data, and then make a response table for the MEA of each factor. When the main effect value of a factor is larger, it means that the factor has a greater impact on the system. On the contrary, the smaller it is, such as Equations (2) and (3).

(2) $${\bar{F}}_{i}=\frac{1}{m}\sum_{j=1}^{m}\eta_{j}$$

(3) $$\Delta F=\max\{{\bar{F}}_{1},{\bar{F}}_{2},{\bar{F}}_{3},\ldots ,{\bar{F}}_{n}\}-\min\{{\bar{F}}_{1},{\bar{F}}_{2},{\bar{F}}_{3},\ldots ,{\bar{F}}_{n}\}$$

where m is the number of level i in the factor row of the orthogonal table, η_j_ is the S/N ratio produced by each j level column, n is the level of the factor.

#### 3.1.4. ANOVA

ANOVA analyzes the contribution of each factor to determine the importance of each factor:

I. Degree of freedom (DOF)
  (1) degrees of freedom for each factor
    (4) $${\mathrm{DOF}}_{\mathrm{factor}}=(\mathrm{level}\;\mathrm{number})-1$$
  (2) total number of degrees of freedom
    (5) $${\mathrm{DOF}}_{\mathrm{total}}=n\times r-1=L-1$$
    where n is the number of experimental groups, r is the number of repeated experiments, and L is the total number of experiments.
  (3) error degrees of freedom
    (6) $${\mathrm{DOF}}_{\mathrm{error}}={\mathrm{DOF}}_{\mathrm{total}}-\sum {\mathrm{DOF}}_{\mathrm{factor}}$$
II. Total sum of squares (SS_T_), the total variation
  (7) $${\mathrm{SS}}_{T}=\sum_{i=1}^{n}{\left(\eta_{i}-{\bar{\eta }}_{i}\right)}^{2}=\sum_{i=1}^{n}{\eta_{i}}^{2}-\mathrm{CF}$$
  where n is the total number of experimental observations, η_i_ is the S/N ratio of each group of experiments, and is the average of overall S/N ratio.

CF is the correction factor, defined as:

(8) $$\mathrm{CF}=\frac{1}{n}\sum_{i=1}^{n}{\left(\eta_{i}\right)}^{2}$$

III. The sum of squares of each factor (SS), the variation of each factor. if a factor has p levels, and each level has m observations, then the sum of squares is:
  (9) $${\mathrm{SS}}_{A}=\frac{1}{m}\left({A_{1}}^{2}+{A_{2}}^{2}+{A_{3}}^{2}+\ldots +{A_{p}}^{2}\right)-\mathrm{CF}$$
IV. Error sum of squares (SSerror):
  (10) $${\mathrm{SS}}_{\mathrm{error}}={\mathrm{SS}}_{T}-\sum {\mathrm{SS}}_{\mathrm{factor}}$$
V. Mean square, MS, the variance:
  (11) $$\mathrm{MS}=\frac{\mathrm{SS}}{{\mathrm{DOF}}_{\mathrm{factor}}}$$
VI. Error mean square (MSE)
  (12) $$\mathrm{MSE}=\frac{{\mathrm{SS}}_{\mathrm{error}}}{{\mathrm{DOF}}_{\mathrm{error}}}$$
VII. F-ratio indicates the relationship between the factor effect and the error variation. When the F value is larger, it means that the factor has a more important influence on the system, and it is used to arrange the important order of the factors.
  (13) $$F=\frac{\mathrm{MS}}{\mathrm{MSE}}$$
VIII. Pure sum of square (SS′)
  (14) $${\mathrm{SS}}_{\mathrm{factor}}^{\prime }={\mathrm{SS}}_{\mathrm{factor}}-{\mathrm{DOF}}_{\mathrm{factor}}\times \mathrm{MSE}$$
IX. Percent contribution (ρ), the relative ability to reduce variation for factors.
  (15) $$\rho =\frac{{\mathrm{SS}}_{\mathrm{factor}}^{\prime }}{{\mathrm{SS}}_{T}}\times 100\%$$

#### 3.1.5. Confidence Interval (CI)

To evaluate each observation value effectively, it is necessary to calculate its CI.

(16) $$CI_{S/N}=\sqrt{F_{\alpha ;1,V_{2}}\times \mathrm{MSE}\times \left(\frac{1}{n_{eff}}+\frac{1}{r}\right)}$$

where $F_{\alpha ;1,V_{2}}$ is the F value with a significant error α, v_2_ is the degree of freedom of the combined error variance, MSE is the combined mean square error, r is the number of confirmation experiments, and n_eff_ is the effective observation value.

(17) $$n_{\mathrm{eff}}=\frac{\mathrm{total}\;\mathrm{number}\;\mathrm{of}\;\mathrm{experiments}}{1+(\mathrm{sum}\;\mathrm{of}\;\mathrm{degrees}\;\mathrm{of}\;\mathrm{freedom}\;\mathrm{for}\;\mathrm{factors}\;\mathrm{to}\;\mathrm{evaluate}\;\mathrm{the}\;\mathrm{mean})}$$

Calculate the 95% confidence interval to verify the validity of the confirmed experimental mean under the predicted optimal parameter conditions, as sown in Equation (18).

(18) $$\overset{⌢}{S}N-CI_{S/N}\leq \mu_{\mathrm{confirmation}}\leq \overset{⌢}{S}N+CI_{S/N}$$

where $\mu_{\mathrm{confirmation}}$ is the mean value of the confirmation experiment.

And

(19) $$\overset{⌢}{S}N=\bar{T}+\sum_{i=1}^{n}(F_{i}-\bar{T})$$

where $\bar{T}$ is the total average of S/N ratio, Fi is the S/N ratio of significant factor level.

### 3.2. PCA [36]

The steps to use PCA are described as follows:

Step 1. List the quality data of each group of experiments, and obtain the S/N ratio of its quality characteristics for PCA.

Step 2. Use Equation (20) to normalize the data of each quality characteristic, so that the data is between 0 and 1

(20) $$X_{\mathrm{normalization}}=\frac{x_{i}\left(j\right)-{\min[x}_{i}\left(j)\right]}{{\max[x}_{i}\left(j)\right]-{\min[x}_{i}\left(j)\right]}$$

Step 3. The normalized data is obtained to obtain the correlation coefficient matrix

(21) $$\rho_{\mathrm{xy}}=\frac{\sum (x_{i}-\bar{x})(y_{i}-\bar{y})}{\sqrt{\sum {\left(x_{i}-\bar{x}\right)}^{2}}\sqrt{\sum {(y_{i}-\bar{y})}^{2}}}$$

where $\rho_{xy}$ is the correlation coefficient of x to y, and $\bar{x}$ is the average value of item x.

Step 4. Use the correlation coefficient matrix to obtain its eigenvalues, which are the principal components, and the corresponding eigenvectors. The variation of the *i*-th principal component is shown in Equation (22).

(22) $$\rho_{i}=\frac{\lambda_{i}}{\sum_{i=1}\lambda_{i}}$$

where $\rho_{i}$ is the variance of the i-th principal component in the total variation, and λ_i_ is the eigenvalue of the i-th principal component.

Step 5: Using the eigenvectors corresponding to the eigenvalues of the principal components and the normalized matrix X, the score of the principal components can be obtained from Equation (22).

(23) $$Y_{i}={\mathrm{XV}}_{i}$$

### 3.3. Data Envelopment Analysis (DEA) [40]

DEA is a fractional mathematical programming technique for evaluating the relative efficiency of decision making unit (DMU) with multiple inputs and multiple outputs. It combines various inputs and various outputs for a DMU into one performance measure (called relative efficiency).

#### 3.3.1. Charnes, Cooper and Rhodes (CCR) Input-Oriented Model

Based on the current output level, discuss how much “input” should be used to be an efficient DMU, and establish an evaluation model for DMU_k_:

(24) $$\mathrm{Maximize}\;\;\;h_{k}=\frac{\sum_{r=1}^{s}\mu_{r}y_{\mathrm{rk}}}{\sum_{i=1}^{m}V_{i}X_{\mathrm{ik}}}$$

(25) $$\begin{array}{ll} \mathrm{Subject}\;\mathrm{to} & \frac{\sum_{r=1}^{s}\mu_{r}y_{\mathrm{rj}}}{\sum_{i=1}^{m}V_{i}X_{\mathrm{ij}}}\leq 1,\;j=1,\cdots \cdots ,n\\ & \mu_{r}\geq 0;\;r=1,\cdots \cdots ,s\\ & v_{i}\geq 0;\;i=1,\cdots \cdots ,m \end{array}$$

where $\mu_{r}$, $v_{i}$ are the weights of the r-th output item and the i-th input item, respectively.

Equation (25) indicates that the “output combination” of any DMU cannot be greater than its “input combination”.

Set $\sum_{i=1}^{m}V_{i}X_{\mathrm{ik}}=1$, Equations (24) and (25) can be changed to Equations (26) and (27).

(26) $$\mathrm{Maximize}\;\;\;\;\;h_{k}=\sum_{r=1}^{s}\mu_{r}y_{\mathrm{rk}}$$

(27) $$\begin{array}{ll} \mathrm{Subject}\;\mathrm{to} & \sum_{i=1}^{m}V_{i}X_{\mathrm{ik}}=1\\ & \sum_{r=1}^{s}\mu_{r}y_{\mathrm{rj}}-\sum_{i=1}^{m}V_{i}X_{\mathrm{ij}}\leq 0,\;j=1,\cdots \cdots ,n\\ & \mu_{r},\;v_{i}\geq 0,\;\forall_{r,i} \end{array}$$

#### 3.3.2. Cross-Efficiency Analysis Model

The cross-evaluation measure was introduced by Sexton, et al. [42]. Let E_oj_ denotes the cross-efficiency of DMU_j_ calculated according to the optimal weights of DMU_o_. For each E_oj_, it is the (weighted output)/(weighted input) obtained by substituting the $u_{\mathrm{ro}}^{*}$ and $v_{io}^{*}$ corresponding to the o-th evaluated unit into the observed value of the j-th evaluated unit, as shown in Equation (28).

(28) $$\begin{array}{cc} E_{\mathrm{oj}}=\frac{\sum_{r=1}^{s}u_{\mathrm{ro}}^{*}y_{\mathrm{rj}}}{\sum_{i=1}^{m}v_{\mathrm{io}}^{*}x_{\mathrm{ij}}} & E_{\mathrm{oj}}\leq 1,\;o\neq j \end{array}$$

This uses DEA in a peer-evaluation instead of a self-evaluation calculated by CCR model.

Let the mean of cross-efficiencies for DMU_j_ expressed as:

(29) $$\begin{array}{cc} e_{j}=\sum_{o\neq j}\frac{E_{\mathrm{oj}}}{(n-1)} & j=1,\cdots \cdots ,n \end{array}$$

The ordinal value is to rank the e_j_ values such that the smallest e_j_ value obtains one whereas the largest e_j_ value gets n.

Let $AOV_{fg}$ is the average of the ordinal values for level g of factor f. From calculating $AOV_{fg}$ value for each factor level. The optimal factor level, $g^{*}$, is chosen as the level that maximizes the value of $AOV_{fg},$ denoted by

(30) $$\begin{array}{cc} g^{*}=\left\{g|\underset{g}{\max}\{{\mathrm{AOV}}_{fg}\right\} & \forall f \end{array}$$

Cross-efficiency maximizes self-evaluation efficiency and minimizes peer-evaluation efficiency.

### 3.4. Materials Analysis

**Figure 5:**
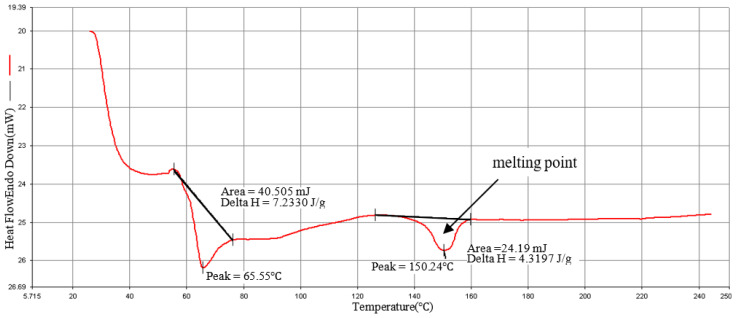
PLA/10%GF (type: GG-0010N) DSC diagram.

**Figure 6:**
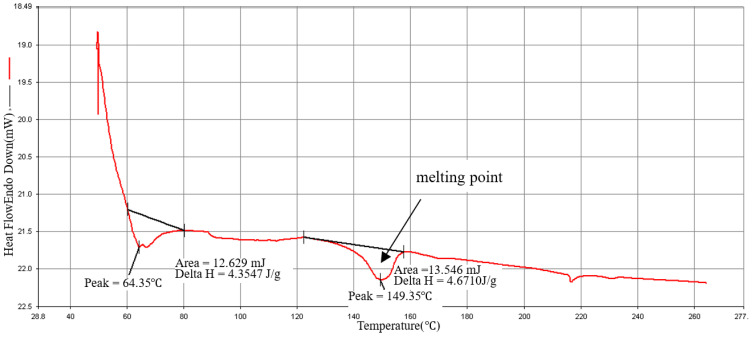
PLA/15%GF (type: GG-0015N) DSC diagram.

**Figure 7:**
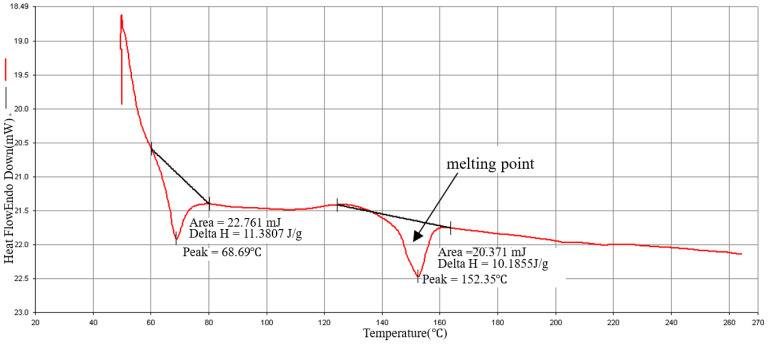
PLA/20%GF type: GG-0020N) DSC diagram.

The DSC is used to measure the melting point of the composites’ material. The sample 2.0 mg is placed in the sample pan. The operating condition rises from 20 °C to 270 °C at a heating rate of 20 °C/min as shown in Figure 5, Figure 6 and Figure 7. The melting point of the material is about 152 °C, which is close to the melting point (155 °C) provided by the manufacturer, so that processing temperature of the composite material should not be lower than this melting point. Verify that the recommended injection temperature provided by the manufacturer is 170 °C~195 °C, which can be used as the melt temperature factor level in the orthogonal table. Kumar and Prakash [43] explained the DSC analysis of pure PLA and composites of PLA. They discussed the thermal characterizations of the composites. There were two peaks at 60.06 °C for glass transition temperature (Tg) and 147.71 °C melt temperature (Tm), with Delta values 0.6354 J/g and 28.2 J/g was observed for pure PLA as explained in literature. When these peak values observed in 20% PLA composites with glass fibers, it was increased to 68.69 °C and 152.35 °C with Delta values 11.387 J/g and 20.371 J/g. Overall, these results explained that PLA composites marks an enhanced thermal behavior and these results are consistent with the literature [4]. The use of other material to synthesize PLA composite raises the polymer breakdown temperature. The differential scanning calorimetry (DSC) curves showed the same behavioral properties as explained in present articles.

### 3.5. Injection Molding Process Parameter Selection

This project is to use the water circulation to cool the injection molding test sample mold. This cooling method is especially suitable for molds with simple shapes and can achieve a uniform cooling effect. By ensuring that the mold is cooled evenly, we can ensure that the quality and dimensions of the product meet the requirements. The parameters that affect the finished workpiece in the injection molding process are speed, temperature, pressure and time [26,28,29,30,31]. Because the speed affects the amount of cavity filler, the temperature affects the shear viscosity of the material, the pressure affects the volumetric shrinkage, and the time depends on the size of the injection molding equipment and the residence time of the material. RTP Company has confirmed that the glass fiber content reinforced polylactic acid compound improves the mechanical properties of polylactic acid. Refer to the machine operation handbook as well, so the glass fiber, so the glass fiber content, melt temperature, injection speed, holding pressure, holding time, and cooling time are set as the control parameters of the injection molding machine. Then the experiments were actually tried out, and the other levels that could result in deviations in the quality of composite material were tried to find, thereby identifying a suitable working range. Finally, for the composite material injection molding processing parameters, the factors that were actually controllable by the injection-molding machine were chosen.

When the temperature is lower than 175 °C, due to high viscosity by the incomplete melting of the material, the nozzle will be stuck. When the temperature is higher than 195 °C, the injection molded test piece will be coked and carbonized, so the processing temperature range is set at 175 °C~195 °C. The control factors and their levels of this experiment are as shown in Table 2.

In this study, the level value of the control factor was applied to the L_18_ (3^6^) orthogonal table for experimental planning. Each group had five test pieces, a total of 90 experimental data. The MEA and ANOVA were used to obtain the optimal process parameters for each quality.

## 4. Experiment results

### 4.1. Experimental Data and Corresponding S/N Ratios

The results for the three iterations of the 18 experiments over 5 iterations with averages, and S/N ratios of five quality characteristics are shown in Table 3.

### 4.2. Single Quality Optimization Analysis

#### 4.2.1. Tensile Strength Test Data Analysis

(1) MEA

**Figure 8:**
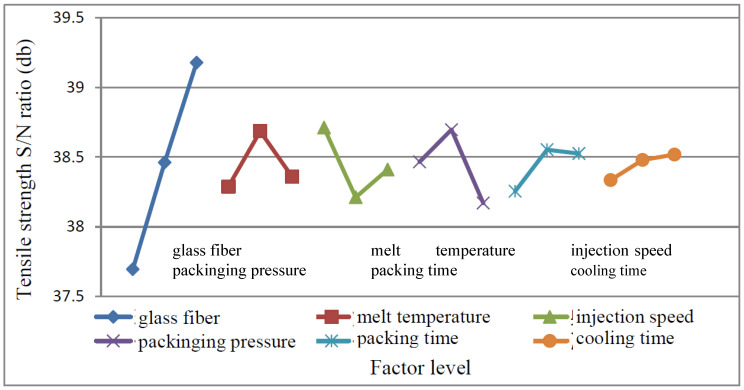
Response graph of Tensile strength.

From the S/N ratio obtained from the experiment as shown in Table 3, the main effect of each control factor is calculated, and the response graph is drawn, as shown in Figure 8. It shows that the best factor level selection is A_3_ (glass fiber 20%), B_2_ (melt temperature: 185 °C), C_1_ (injection speed: 40 mm/s), D_2_ (packing pressure: 60 MPa), E_2_ (packing pressure Time: 1 s), F_3_ (cooling time: 20 s). According to the amount of change in the graph, it is judged that the control factor A has the greatest influence on this quality characteristic, followed by D, C, B, E, and F.

(2) ANOVA

From ANOVA, the larger the F value is, the greater the contribution is, and it is expressed as a significant factor. Generally, the F value less than 5 is regarded as a factor with a relatively low contribution and its error is incorporated into the combined error. The ANOVA of tensile strength as shown in Table 4. The most significant factor is A (glass fiber), followed by D (packing pressure), C (injection speed), B (melt temperature).

In order to effectively evaluate each observation value and calculate its confidence interval, the expected mean value of the calculation confirmation experiment is:

(31) $${\mathrm{CI}}_{S/N}=\sqrt{F_{{\alpha ;1,V}_{2}}\times \mathrm{MSE}\times \left(\frac{1}{n_{\mathrm{eff}}}+\frac{1}{r}\right)}=\sqrt{5.12\times 0.071785\times \left(\frac{1}{\frac{18}{1+8}}+\frac{1}{5}\right)}=0.5072$$

Its $\overset{⌢}{S}N=39.93545\;\mathrm{db}$, 95% confidence interval is 39.4282 ≤ μ_confirmation_ ≤ 40.4427.

#### 4.2.2. Hardness Test Data Analysis

(1) MEA

**Figure 9:**
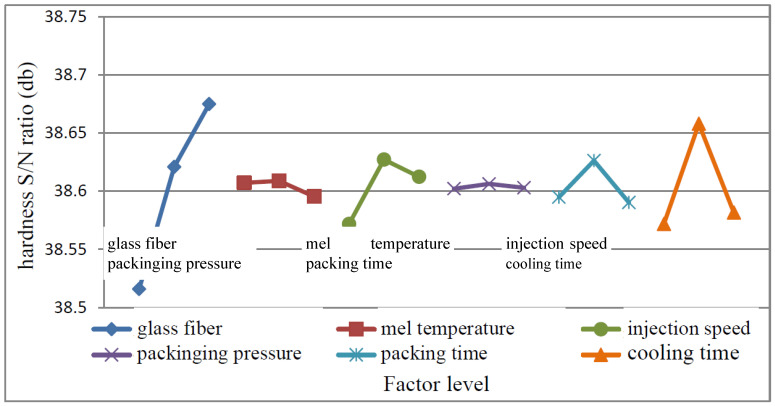
Hardness factor response graph.

From the S/N ratio obtained from the experiment as shown in Table 4, the main effect of each control factor was calculated, and the response graph was drawn as shown in Figure 9. It shows that the optimal factor levels are A_3_ (glass fiber: 20%), B_2_ (melt temperature: 185 °C), C_2_ (injection speed: 60 mm/s), D_2_ (packing pressure: 60 MPa), E_2_ (packing time: 1 s), F_2_ (cool time: 15 s). According to the amount of change in the graph, it can be judged that the control factor A has the greatest influence on this quality characteristic, followed by F, C, E, B, and D.

(2) ANOVA

It can be seen from Table 5 that the most significant factor is A (glass fiber), followed by F (cooling time), and C (injection speed). Since the F values of E, B, and D are less than 5, the contribution is considered relatively low factor into the combined error.

In order to effectively evaluate each observation value and calculate its confidence interval, the expected mean value of the calculation confirmation experiment is:

(32) $${\mathrm{CI}}_{S/N}=\sqrt{F_{{\alpha ;1,V}_{2}}\times \mathrm{MSE}\times \left(\frac{1}{n_{\mathrm{eff}}}+\frac{1}{r}\right)}=\sqrt{4.84\times 0.000837\times \left(\frac{1}{\frac{18}{1+6}}+\frac{1}{5}\right)}=0.0488$$

Its $\overset{⌢}{S}N=39.93545\;\mathrm{db}$, 95% confidence interval is 38.7035 ≤ μ_confirmation_ ≤ 38.8011.

#### 4.2.3. Impact Strength Test Data Analysis

(1) MEA

**Figure 10:**
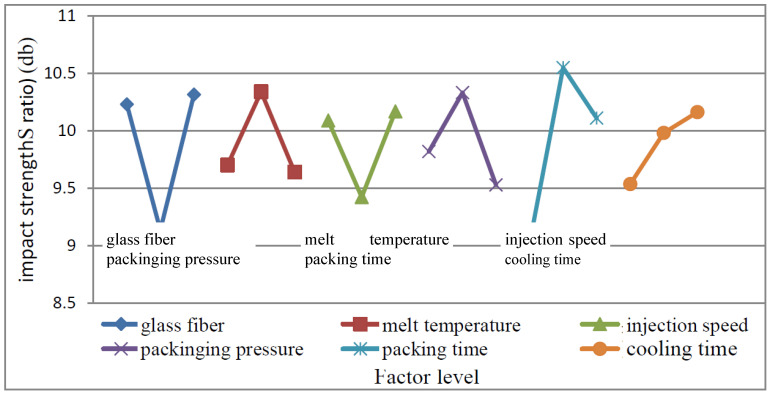
Impact strength response graph.

From the S/N ratio obtained from the experiment as shown in Table 3, the main effect of each control factor is calculated, and the response graph is drawn, as shown in Figure 10. It shows that the best factor levels are A_3_ (glass fiber 20%), B_2_ (melt temperature 185 °C), C_3_ (injection speed 80 mm/s), D_2_ (packing pressure 60 MPa), E_2_ (packing time 1 s), F_3_ (cooling time 20 s). According to the variation of the graph, it can be observed that the control factor E has the greatest influence on this quality characteristic, followed by A, D, C, B, and F.

(2) ANOVA

From ANOVA Table 6, it shows that the most significant factor is E (packing time), followed by A (glass fiber), D (packing pressure), C (injection speed), and B (melt temperature).

In order to effectively evaluate each observation value and calculate its confidence interval, the expected mean value of the calculation confirmation experiment is:

(33) $${\mathrm{CI}}_{S/N}=\sqrt{F_{{\alpha ;1,V}_{2}}\times \mathrm{MSE}\times \left(\frac{1}{n_{\mathrm{eff}}}+\frac{1}{r}\right)}=\sqrt{5.59\times 0.269678\times \left(\frac{1}{\frac{18}{1+10}}+\frac{1}{5}\right)}=1.1058$$

Its $\overset{⌢}{S}N=12.13171\;\mathrm{db}$, 95% confidence interval is 11.0259 ≤ μ_confirmation_ ≤ 13.2375.

#### 4.2.4. Bending Strength Experiment Data Analysis

(1) MEA

**Figure 11:**
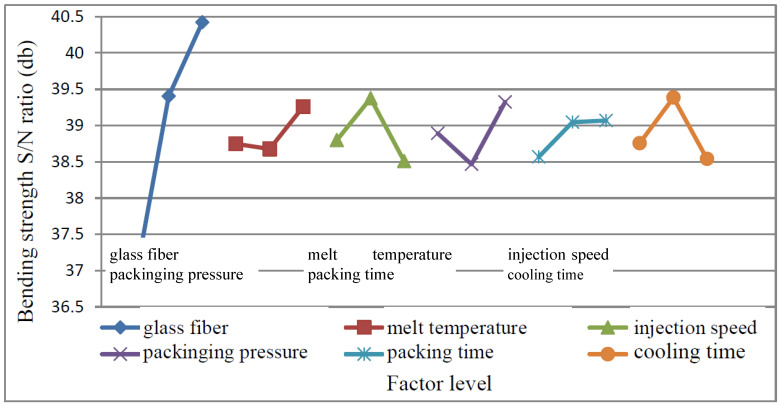
Bending factor response graph.

From the S/N ratio obtained from the experiment as shown in Table 3, the main effect of each control factor was calculated, and the response graph was drawn, as shown in Figure 11. It shows that the optimal factor level selection is A_3_ (glass fiber is 20%), B_3_ (melt temperature 195 °C), C_2_ (injection speed 60 mm/s), D_3_ (holding pressure 70 MPa), E_3_ (holding time 1.5 s), F_2_ (cooling time 15 s). According to the variation of the graph, it can be observed that the control factor A has the greatest influence on this quality characteristic, followed by C, D, F, B, and E.

(2) ANOVA

From ANOVA Table 7, it shows that the most significant factor is A (glass fiber), followed by C (injection speed), D (packing pressure), and F (cooling time).

In order to effectively evaluate each observation value and calculate its confidence interval, the expected mean value of the calculation confirmation experiment is:

(34) $${\mathrm{CI}}_{S/N}=\sqrt{F_{{\alpha ;1,V}_{2}}\times \mathrm{MSE}\times \left(\frac{1}{n_{\mathrm{eff}}}+\frac{1}{r}\right)}=\sqrt{5.12\times 0.344485\times \left(\frac{1}{\frac{18}{1+8}}+\frac{1}{5}\right)}=1.1111$$

Its $\overset{⌢}{S}N=41.82273\;\mathrm{db}$, 95% confidence interval is 40.7116 ≤ μ_confirmation_ ≤ 42.9339.

### 4.3. Multiple-Quality Optimization Analysis

In this section, the Taguchi method is used in conjunction with PCA and DEA to obtain multiple quality optimization process parameters.

#### 4.3.1. PCA

Step 1. From Table 4, normalize the S/N ratio data of each quality according to Equation (20), as shown in Table 8.

Step 2. Calculate the correlation coefficient matrix of the normalized data according to Equation (21), as shown in Table 9.

Step 3. Use the correlation coefficient matrix to calculate the eigenvalues and the eigenvectors, such as in Table 10 and Table 11; According to Equation (22), the variation of each principal component in the total variation is obtained.

Step 4. Combine the normalized data in Table 8 and the eigenvectors in Table 11, and calculate the total scores of the principal components according to Equation (23), as shown in Table 12.

Step 5. Multi-quality optimal parameter combination. The principal component total scores corresponding to the various control factors are shown in Table 13.

The best combination of parameters is A_2_ (glass fiber: 15%), B_2_ (melt temperature: 185 °C), C_1_ (injection speed: 40 mm/s), D_2_ (packing pressure: 60 MPa), E_1_ (packing time: 0.5 s), F_3_ (cooling time: 20 s).

#### 4.3.2. DEA

Step 1. According to Equations (26) and (27), the relative efficiency is calculated from Table 4, as shown in Table 14 and the optimal weight of output and input is shown in Table 15.

Step 2. According to Equation (28), Table 14 and Table 15 are sorted by cross efficiency, and calculate the level value of the corresponding control factor in the orthogonal table, as shown in Table 16.

Table 16 shows that the best parameter combinations are A_3_ (glass fiber: 20%), B_2_ (melt temperature: 185 °C), C_3_ (injection speed: 80 mm/s), D_2_ (packing pressure: 60 MPa), E_2_ (packing time: 1 s), F_2_ (cooling time: 15 s).

## 5. Discussions

### 5.1. S/N Ratio Additive Model

Use the S/N ratio addition model to predict the S/N ratio of the best combination to verify the rationality of the confirmation experimental data.

(1) PCA

The best combined S/N ratio addition model of PCA is shown in Table 17. For example, the S/N ratio addition model of the strength quality of the optimal factor level combination is calculated as follows:

(35) $$\begin{array}{l} {\overset{⌢}{\rho }}_{A_{2}B_{2}C_{1}D_{2}E_{1}F_{3}}=38.44403+\left(38.4606-38.44403\right)+\left(38.68471-38.4403\right)+\left(38.71073-38.44403\right)\\ +\left(38.69628-38.44403\right)+\left(38.25426-38.44403\right)+\left(38.51775-38.44403\right)=39.10419 \end{array}$$

Similarity, the best combined S/N ratio addition model of DEA is shown in Table 18.

(2) DEA

Use the S/N ratio addition model to predict the S/N ratio of the best combination to verify the rationality of the confirmation experimental data.

(36) $$\begin{array}{l} \overset{⌢}{\rho }A_{3}B_{2}C_{3}D_{2}E_{2}F_{2}=38.44403+\left(39.17581-38.44403\right)+\left(38.68471-38.44403\right)+\left(38.4098-38.44403\right)\\ +\left(38.69628-38.44403\right)+\left(38.55264-38.44403\right)+\left(38.47925-38.44403\right)=39.77835 \end{array}$$

Similarity, the prediction of all qualities is shown in Table 18.

### 5.2. S/N Ratio Additive Model Comparison

From Table 19, it can be seen that the S/N ratio of the optimal factor level combination of DEA in total qualities can be improved by 5.537101 db compared with the PCA expectation, so it can be predicted that the optimal factor level combination of multiple qualities is A_3_B_2_C_3_D_2_E_2_F_2_.

### 5.3. Confirmation Experiment and Comparison

The best processing parameters are actually processed the test pieces on the injection molding machine, and carry out the confirmation experiment. Each group of experiments is performed 5 times, as shown in Table 20 and Table 21, and the comparison is as follows.

(1) The S/N ratio of the confirmation experiment of the two methods are similar to those predicted by the S/N ratio additive model.
(2) The average confirmation experiment data of DEA: tensile strength 95.03775 MPa, hardness 86.52 Shore D, impact strength 4.4408 J/cm^2^, bending strength 119.889 MPa.
(3) The average confirmation experiment data of PCA: tensile strength 94.03601 MPa, hardness 86.28 Shore D, impact strength 3.285046 J/cm^2^, bending strength 98.21989 MPa.
(4) The Taguchi method combined with DEA, the obtained optimal combination of process parameters has the characteristics of better and multi-quality considerations.

The comparison of the multiple quality confirmation experiment group with single quality best experiment group from Taguchi experiment is shown in Table 22. It is observed that the optimal combination of process parameters obtained from DEA can meet the goal of the best multi-quality optimization.

## 6. Conclusions

In this paper, polylactide with glass fiber composites were synthesized via injection molding process and optimized with process parameters. First, the Taguchi orthogonal table is used to conduct experiments, and the optimal parameters of the single-quality process are obtained through MEA and ANOVA. Then, the PCA and DEA was combined to get the optimal process parameters for multiple qualities, and five confirmation experiments are carried out respectively to verify the ability of multi-quality consideration. The optimal process conditions are found to be glass fiber addition of 20%, melt temperature of 185 °C, injection speed of 80 mm/s, holding pressure of 60 MPa, retaining time of 1 s, and cooling time of 15 s. The associated mechanical properties are tensile strength of 95.04 MPa, hardness of 86.52 Shore D, impact strength of 4.4408 J/cm^2^, and bending strength of 4.4408 J/cm^2^. This research successfully boosts several properties of the PLA/GF composite. The composite material used in this study, the degradability of polylactic acid and the recyclability of glass fiber can reduce environmental pollution, and the mechanical properties can also be enhanced at the same time, that non-decomposable plastic materials cannot achieve.

## Figures and Tables

**Table 1:** PLA/GF material properties.

	Type	GG-0010N	GG-0015N	GG-0020N
Category		TY11512706	TY11512707	TY11512708
Raw material properties		Ratio	Ratio	Ratio
Filling contents (%)		10	15	20
Mold shrinkage (%)		0.08	0.07	0.055
Melting point (°C)		155	155	155
Specific weight		1.302	1.352	1.373

**Table 2:** Injection molding machine control factors and their level values.

	Factor	A	B	C	D	E	F
Level		GF (%)	Melt Temperature (°C)	Injection Speed (mm/s)	Packing Pressure (MPa)	Packing Time (s)	Cololing Time (s)
1		10	175	40	50	0.5	10
2		15	185	60	60	1	15
3		20	195	80	70	1.5	20

**Table 3:** L_18_ orthogonal array of experimental data.

Exp. No.	Tensile Strength		Shore Hardness		Impact Strength		Bending Strength	
	Mean (Mpa)	S/N Ratio (db)	Mean (Shore D)	S/N Ratio (db)	Mean (Mpa)	S/N Ratio (db)	Mean (Mpa)	S/N Ratio (db)
1	73.52	37.32	83.52	38.43	2.84	9.05	66.81	36.49
2	79.38	37.99	85.36	38.62	3.82	11.64	73.57	37.33
3	74.35	37.42	84.16	38.50	3.30	10.36	69.94	36.89
4	89.81	39.06	85.32	38.62	3.31	10.41	81.85	38.26
5	79.97	38.05	85.08	38.59	2.72	8.65	104.78	40.40
6	81.27	38.19	85.8	38.66	2.67	8.47	94.10	39.46
7	90.01	39.08	86.64	38.75	3.00	9.51	114.29	41.15
8	94.58	39.51	85.52	38.64	3.43	10.64	89.02	38.98
9	90.93	39.17	85.28	38.61	3.22	10.09	113.04	41.06
10	74.18	37.40	84.96	38.58	3.41	10.56	76.23	37.63
11	84.20	38.50	83.6	38.44	3.68	11.29	65.02	36.25
12	75.22	37.52	84.2	38.50	2.65	8.45	66.95	36.51
13	76.84	37.71	85.16	38.60	2.42	7.59	93.94	39.45
14	86.97	38.78	85.44	38.63	3.13	9.90	85.10	38.59
15	88.53	38.94	85.12	38.60	3.08	9.77	102.68	40.22
16	90.59	39.13	85.56	38.64	3.59	11.06	94.10	39.46
17	91.77	39.25	86.24	38.71	3.13	9.90	105.74	40.47
18	88.04	38.89	85.88	38.67	3.43	10.67	117.27	41.38

**Table 4:** ANOVA of tensile strength.

Source of Variance	DOF	SS	MS	F-Ratio	SS′	Contribution (%)
A	2	6.574942	3.287471	78.8653	6.491573	69.43184
B	2	0.536076	0.268038	6.430137	0.452706	4.842008
C	2	0.758098	0.379049	9.093256	0.674729	7.216688
D	2	0.834386	0.417193	10.00831	0.751016	8.032637
E	2	0.326381	0.163191	3.91489	0.243012	2.599183
F	2	0.111256	0.055628	1.334499	0.027887	0.29827
Error	5	0.208423	0.041685	-	0.708639	7.579378
Combined error	9	0.646061	0.071785	-	0.979538	10.47683
Total	17	9.349562	-	-	9.349562	100%

**Table 5:** Hardness ANOVA table.

Source of Variance	DOF	SS	MS	F-Ratio	SS	Contribution (%)
A	2	0.078381	0.03919	50.17795	0.076819	61.88925
B	2	0.000645	0.000322	0.41269	−0.00092	−0.73912
C	2	0.009879	0.004939	6.324327	0.008317	6.700536
D	2	0.0000583	0.0000292	0.037343	−0.0015	−1.21148
E	2	0.004601	0.0023	2.945448	0.003039	2.448299
F	2	0.026654	0.013327	17.06346	0.025092	20.21547
Error	5	0.003905	0.000781	-	0.013277	10.69704
Combined error	11	0.009209	0.000837	-	0.013895	11.19475
Total	17	0.124123	-	-	0.124123	100%

**Table 6:** ANOVA of impact strength.

Source of Variation	DOF	SS	MS	F-Ratio	SS^′^	Contribution (%)
A	2	5.194863	2.597431	20.26545	4.938522	24.29646
B	2	1.806817	0.903408	7.048492	1.550476	7.628006
C	2	1.988054	0.994027	7.755511	1.731713	8.519656
D	2	2.013387	1.006693	7.854334	1.757046	8.644286
E	2	7.435231	3.717616	29.00525	7.17889	35.31859
F	2	1.246892	0.623446	4.864195	0.990551	4.873297
error	5	0.640852	0.12817	-	2.178897	10.7197
combined error	7	1.887744	0.269678	-	3.169448	15.593
Total	17	20.3261	-	-	20.3261	100%

**Table 7:** Bending ANOVA table.

Source of Variation	DOF	SS	MS	F-Ratio	SS′	Contribution (%)
A	2	40.49939	20.2497	109.8102	40.13058	79.54693
B	2	1.217893	0.608946	3.302198	0.84908	1.683048
C	2	2.333172	1.166586	6.326172	1.96436	3.893759
D	2	2.307224	1.153612	6.255815	1.938411	3.842324
E	2	0.960443	0.480222	2.604149	0.591631	1.172732
F	2	2.208777	1.104389	5.988887	1.839965	3.647182
error	5	0.922032	0.184406	-	3.134908	6.214022
combined error	9	3.100368	0.344485	-	4.575619	9.069802
Toatl	17	50.44894	-	-	50.44894	100%

**Table 8:** Normalization of quality data.

Item	Normalization			
Exp. No.	Tensile Strength (db)	Hardness (db)	Impact Strength (db)	Bending Strength (db)
1	0	0	0.360288	0.046529
2	0.305514	0.595101	1	0.209861
3	0.043814	0.211606	0.68312	0.124504
4	0.796652	0.583344	0.695165	0.39112
5	0.335628	0.506342	0.260825	0.80949
6	0.398977	0.734969	0.217705	0.626838
7	0.805877	1	0.474639	0.955724
8	1	0.646452	0.751809	0.532859
9	0.845459	0.570436	0.616277	0.937851
10	0.036197	0.46859	0.733954	0.269505
11	0.53995	0.029864	0.912518	0
12	0.092801	0.223351	0.212693	0.050202
13	0.177234	0.531425	0	0.624045
14	0.66961	0.621067	0.5699	0.456059
15	0.740098	0.519641	0.538667	0.77504
16	0.830143	0.656811	0.855435	0.625622
17	0.88272	0.874393	0.570041	0.823749
18	0.717439	0.760387	0.759501	1

**Table 9:** Correlation coefficient matrix.

Correlation Coefficient	Tensile Strength	Hardness	Impact Strength	Bending Strength
tensile strength	1	0.637712	0.364163	0.631945
hardness	0.637712	1	0.025061	0.804754
impact strength	0.364163	0.025061	1	−0.15557
bending strength	0.631945	0.804754	−0.15557	1

**Table 10:** Eigenvalues and variances.

Principal Component	Eigenvalues	Variance (%)	Variance Accumulation (%)
1	2.3972	59.9315	59.9315
2	1.1729	29.32323	89.25473
3	0.2764	6.910173	96.1649
4	0.1534	3.835096	100

**Table 11:** Eigenvectors.

Principal Component Eigenvalue	Eigenvector			
	PC1	PC2	PC3	PC4
tensile strength	0.3327	0.6983	−0.3061	0.555
hardness	0.4779	−0.6381	0.1317	0.5891
impact strength	−0.2964	−0.3228	−0.8943	0.0906
bending strength	−0.7571	0.0304	0.2987	0.5803

**Table 12:** The principal component scores.

PC No.	PC1	PC2	PC3	PC4	MPCI
1	−0.14202	−0.11489	−0.30831	0.059643	−0.13782
2	−0.06924	−0.48281	−0.84676	0.732517	−0.21349
3	−0.18104	−0.32116	−0.55927	0.283114	−0.23046
4	0.04166	−0.02844	−0.67189	1.075738	0.011456
5	−0.33653	−0.14831	−0.02751	0.977937	−0.20957
6	−0.05513	−0.2416	−0.03279	1.037881	−0.06634
7	−0.11825	−0.19952	−0.25397	1.633971	−0.08426
8	0.01538	0.059314	−0.73414	1.313157	0.026238
9	−0.33882	0.055965	−0.45467	1.405343	−0.16417
10	−0.18560	−0.50246	−0.52524	0.519025	−0.27496
11	−0.07656	0.06343	−0.97741	0.399939	−0.07948
12	0.03657	−0.14485	−0.17421	0.231483	−0.02372
13	−0.15953	−0.19637	0.20214	0.77356	−0.10956
14	0.00539	−0.09881	−0.49661	1.053788	−0.01965
15	−0.25188	0.034907	−0.40833	1.215434	−0.12232
16	−0.13713	−0.09654	−0.74575	1.288207	−0.11262
17	−0.08107	−0.10051	−0.41878	1.534682	−0.04814
18	−0.38013	−0.19898	−0.49999	1.495233	−0.26337

Notes: PC: principal component. MPCI: multiple performance characteristic index.

**Table 13:** Total scores of the principal component.

	Factor	A	B	C	D	E	F
Level							
1		−0.15999	−0.11796	−0.09008	−0.10849	−0.05989	−0.11126
2		−0.08600	−0.09068	−0.15066	−0.07241	−0.15403	−0.13492
3		−0.10772	−0.14506	−0.11297	−0.17281	−0.13979	−0.10753
Optimal combination		A_2_B_2_C_1_D_2_E_1_F_3_					

**Table 14:** The relative efficiency of each DMUj.

DMU_j_	Input	Output				CCR Relative Efficiency
	x_1j_	Tensile Strength (y_1j_)	Hardness (y_2j_)	Impact Strength (y_3j_)	Bending Strength (y_4j_)	E_O_
DMU_1_	1	73.52477	83.52	2.8476	66.81545	0.963989
DMU_2_	1	79.38108	85.36	3.828677	73.57561	1
DMU_3_	1	74.35877	84.16	3.3012	69.94087	0.977845
DMU_4_	1	89.81108	85.32	3.317569	81.85909	0.991989
DMU_5_	1	79.97769	85.08	2.720092	104.784	0.981994
DMU_6_	1	81.27175	85.8	2.672708	94.10377	0.990305
DMU_7_	1	90.01753	86.64	3.008923	114.2964	1
DMU_8_	1	94.58278	85.52	3.433446	89.02934	1
DMU_9_	1	90.93708	85.28	3.220646	113.0414	1
DMU_10_	1	74.18635	84.96	3.417077	76.23644	0.988585
DMU_11_	1	84.20733	83.6	3.680062	65.02789	0.991002
DMU_12_	1	75.22981	84.2	2.656123	66.95524	0.971837
DMU_13_	1	76.84382	85.16	2.421138	93.94801	0.982918
DMU_14_	1	86.97903	85.44	3.138369	85.10043	0.989086
DMU_15_	1	88.53637	85.12	3.089477	102.6813	0.985056
DMU_16_	1	90.59795	85.56	3.597569	94.10114	1
DMU_17_	1	91.77599	86.24	3.138585	105.7453	1
DMU_18_	1	88.04105	85.88	3.4328	117.2763	1

**Table 15:** The optimal weight of each DMUj.

DMU_j_	Input	Output			
	v^*^_1j_	u^*^_1j_	u^*^_2j_	u^*^_3j_	u^*^_4j_
DMU_1_	1	0	0.011542	0	0
DMU_2_	1	0.0000462	0.010776	0.019273	0.0000372
DMU_3_	1	0	0.010948	0.017094	0
DMU_4_	1	0.000828	0.010068	0.017654	0
DMU_5_	1	0	0.011542	0	0
DMU_6_	1	0	0.011542	0	0
DMU_7_	1	0.003315	0.006394	0.021896	0.000715
DMU_8_	1	0.006234	0.001792	0.04055	0.001324
DMU_9_	1	0.005772	0.002066	0.046761	0.001311
DMU_10_	1	0	0.010948	0.017094	0
DMU_11_	1	0.003771	0	0.18301	0
DMU_12_	1	0	0.011542	0	0
DMU_13_	1	0	0.011542	0	0
DMU_14_	1	0.000208	0.010654	0.019339	0
DMU_15_	1	0.000208	0.010654	0.019339	0
DMU_16_	1	0.001158	0.009406	0.021217	0.000149
DMU_17_	1	0.003616	0.005963	0.02334	0.000762
DMU_18_	1	0.000841	0.009809	0.020768	0.000105

**Table 16:** DEA Cross-efficiency sorting corresponds to the control factor level.

	Factor	A	B	C	D	E	F
Level							
1		5.3	9.0	9.8	9.2	7.5	8.0
2		7.7	10.8	8.5	11.7	11.2	10.5
3		15.5	8.7	10.2	7.7	9.8	10.0
Optimal combination		A_3_B_2_C_3_D_2_E_2_F_2_					

**Table 17:** The best combined S/N ratio addition model of PCA.

Best Combination	Tensile Strength	Hardness	Impact Strength	Bending Strength
A_2_	38.4606	38.62083	9.135085	39.40227
B_2_	38.68471	38.6089	10.33989	38.67565
C_1_	38.71073	38.57181	10.08841	38.79719
D_2_	38.69628	38.60631	10.33224	38.46438
E_1_	38.25426	38.59489	9.020387	38.56648
F_3_	38.51775	38.58169	10.16217	38.53941
${\overset{⌢}{\rho }}_{A_{2}B_{2}C_{1}D_{2}E_{1}F_{3}}$	39.10419	38.56534	9.611899	38.98086

**Table 18:** The best combined S/N ratio addition model of DEA.

Optimal Combination	Tensile Strength	Hardness	Impact Strength	Bending Strength
A_3_	39.17581	38.67478	10.31462	40.42158
B_2_	38.68471	38.6089	10.33989	38.67565
C_3_	38.4098	38.61244	10.1688	38.50768
D_2_	38.69628	38.60631	10.33224	38.46438
E_2_	38.55264	38.62627	10.54918	39.04502
F_2_	38.47925	38.65794	9.981667	39.38358
${\overset{⌢}{\rho }}_{A_{3}B_{2}C_{3}D_{2}E_{2}F_{2}}$	39.77835	38.76755	12.22012	41.03337

**Table 19:** DEA addition model improvement.

	Method	DEA S/N (db)	PCA S/N (db)	Improvement S/N (db)
Quality				
tensile strength		39.77835	39.10419	0.67416
hardness		38.76755	38.56534	0.20221
impact strength		12.22012	9.611899	2.608221
bending strength		41.03337	38.98086	2.05251

**Table 20:** PCA’s confirmation experiment.

	Group	1	2	3	4	5	Average	LTB S/N (db)
Quaty								
tensile strength		94.14074	93.86753	93.91307	94.18627	94.07243	94.03601	39.46586
hardness		86.4	86.2	86.2	86.2	86.4	86.28	38.71819
impact strength		3.284615	3.284615	3.449385	3.284615	3.122	3.285046	10.31786
bending strength		97.20731	98.21989	98.21989	98.21989	99.23247	98.21989	39.84344

**Table 21:** PCA’s confirmation experiment.

	Group	1	2	3	4	5	Average	LTB S/N (db)
Quality								
tensile strength		94.52777	95.05141	95.37014	95.55228	94.68714	95.03775	39.5577
hardness		86.2	86.8	86.6	87	86	86.52	38.74209
impact strength		4.358308	4.523077	4.441231	4.523077	4.358308	4.4408	12.94564
bending strength		119.484	120.4966	120.4966	119.484	119.484	119.889	41.57537

**Table 22:** The comparison of multiple quality confirmation experiment group with single quality best experiment group.

	Quality	Tensile Strength (MPa)	Hardness (Shore D)	Impact Strength (J/cm^2^)	Bending Strength (MPa)
Group					
PCA confirmation experimental group		94.03601	86.28	3.285046	98.21989
DEA confirmation experimental group		95.03775	86.52	4.4408	119.889
Taguchi group 8		94.58278	85.52	3.433446	89.02934
Taguchi group 7		90.01753	86.64	3.008923	114.2964
Taguchi group 2		79.38108	85.36	3.828677	73.57561
Taguchi Group 18		88.04105	85.88	3.4328	117.2763

## Data Availability

The data presented in this study are available on request.
